# Patient-individualized resection planning in liver surgery using 3D print and virtual reality (i-LiVR)—a study protocol for a prospective randomized controlled trial

**DOI:** 10.1186/s13063-022-06347-0

**Published:** 2022-05-13

**Authors:** Tobias Huber, Laura Isabel Hanke, Christian Boedecker, Lukas Vradelis, Janine Baumgart, Stefan Heinrich, Fabian Bartsch, Jens Mittler, Alicia Schulze, Christian Hansen, Florentine Hüttl, Hauke Lang

**Affiliations:** 1 Department of General, Visceral and Transplant Surgery, University Medical Center Mainz, Mainz, Germany; 2 Institute of Medical Biostatistics, Epidemiology and Informatics (IMBEI), University Medical Center Mainz, Mainz, Germany; 3 Institute of Simulation and Graphics, Faculty of Informatics, University Magdeburg, Magdeburg, Germany

**Keywords:** Liver surgery, 3D print, Liver resection planning, Virtual reality, Head-mounted display, Surgical training

## Abstract

**Background:**

A multitude of different diseases—benign and malign—can require surgery of the liver. The liver is an especially challenging organ for resection planning due to its unique and interindividually variable anatomy. This demands a high amount of mental imagination from the surgeon in order to plan accordingly - a skill, which takes years of training to acquire and which is difficult to teach. Since the volume of the functional remnant liver is of great importance, parenchyma sparing resections are favoured. 3D reconstructions of computed tomography imaging enable a more precise understanding of anatomy and facilitate resection planning. The modality of presentation of these 3D models ranges from 2D monitors to 3D prints and virtual reality applications.

**Methods:**

The presented trial compares three different modes of demonstration of a 3D reconstruction of CT scans of the liver, which are 3D print, a demonstration on a regular computer screen or using a head-mounted virtual reality headset, with the current gold standard of viewing the CT scan on a computer screen. The group size was calculated with *n*=25 each. Patients with major liver resections in a laparoscopic or open fashion are eligible for inclusion. Main endpoint is the comparison of the quotient between planned resection volume and actual resection volume between these groups. Secondary endpoints include usability for the surgical team as well as patient specifics and perioperative outcome measures and teaching issues.

**Discussion:**

The described study will give insight in systematic planning of liver resections and the comparison of different demonstration modalities of 3D reconstruction of preoperative CT scans and the preference of technology. Especially teaching of these demanding operations is underrepresented in prior investigations.

**Trial registration:**

Prospective trials registration at the German Clinical Trials register with the registration number DRKS00027865. Registration Date: January 24, 2022.

**Supplementary Information:**

The online version contains supplementary material available at 10.1186/s13063-022-06347-0.

## Administrative information

Note: the numbers in curly brackets in this protocol refer to SPIRIT checklist item numbers. The order of the items has been modified to group similar items (see http://www.equator-network.org/reporting-guidelines/spirit-2013-statement-defining-standard-protocol-items-for-clinical-trials/).

**Table:** 

Title {1}	Patient-individualized resection planning in liver surgery using 3D print and virtual reality (VR) – a prospective randomized controlled study
Trial registration {2a and 2b}	Prospective trials registration at the German Clinical Trials register with the registration number DRKS00027865, registration confirmed 24^th^ of January 2022, https://www.drks.de/drks_web/
Protocol version {3}	Version 4.0, dated 15^th^ of November 2021
Funding {4}	The study is supported by a German Research Foundation (DFG) grant over 188.200€ (project number: 490729183)
Author details {5a}	Tobias Huber^1^, Laura Isabel Hanke^1^, Christian Boedecker^1^, Lukas Vradelis^1^, Janine Baumgart^1^, Stefan Heinrich^1^, Fabian Bartsch^1^, Jens Mittler ^1^, Alicia Schulze^2^, Christian Hansen^3^, Florentine Hüttl^1^, Hauke Lang^1^ ^1^Department of General, Visceral and Transplant Surgery, University Medical Center Mainz ^2^ Institute of Medical Biostatistics, Epidemiology and Informatics (IMBEI), University Medical Center Mainz ^3^Institute of Simulation and Graphics, Faculty of Informatics, University Magdeburg
Name and contact information for the trial sponsor {5b}	University Medical Center of the Johannes Gutenberg-University Mainz Langenbeckstrasse 1 55131 Mainz, Germany
Role of sponsor {5c}	The sponsor and funders have no role in the design of the study, collection, analysis or interpretation of the data, neither in the writing of the manuscript. The trial is designed and will be conducted, analysed, interpreted and written by the afore mentioned study team.

## Introduction

### Background and rationale {6a}

Every year, about 80,000 patients are treated in German hospitals for primary hepatic malignancies or metastases of the liver. Approximately 30,000 of those undergo liver resection each year [1]. Resectability of a liver tumour is determined by technical and functional aspects: the tumour needs to be removed with a sufficient resection margin while maintaining a future liver remnant of sufficient size, which is estimated to be 25% of the staring volume [2]. Since liver function cannot be artificially replaced the risk of postoperative liver failure after liver resection is estimated to be 6–23% depending on the extent of resection [3]. Liver surgery is challenging and requires accurate planning due to the liver’s unique anatomical structure as a parenchymatous organ and its division into eight anatomical segments. Each liver segment is supplied with blood by an artery and a portal vein and drained by the hepatic veins as well as bile being drained by the bile duct [4]. The anatomical division into liver segments is highly variable from patient to patient and not visible on the liver surface [5]. Due to this high interpatient variability liver resections need to be individualized and resection planning prior to the operation date is vital. Usually, this planning step is conducted by the surgeon using a contrast-enhanced CT scan or MRI of the Liver and its blood vessels. The two-dimensional pictures need to be transformed into a three-dimensional model threaded with blood vessels and bile ducts by the surgeons power of imagination. This skill set is integral to hepatobiliary surgery and demands years of training [6]. It is a skill, which is not only demanding to learn but also challenging to teach. In order to facilitate easier planning and teaching, computer-based procedures to obtain a three-dimensional model of the liver using a pre-existing CT scan have been developed [7, 8]. This is an individualized process and thus takes the interpatient anatomical variabilities into account [9]. These 3D models allow for planning of the operation and calculation of the resection volume, usually using an interactive PDF displayed on a conventional two-dimensional computer screen [10]. The virtual 3D model of the liver can be rotated and zoomed in as needed in the program. A second option of displaying this three-dimensional data set is a 3D print, in which the liver tissue is displayed in transparent plastic enabling blood vessels, bile duct, tumour and cysts to be visible from the outside [11, 12]. The surgeon can pick up the model and rotate it in her or his hands and plan the resection accordingly. A third modality of displaying a 3D reconstruction of the liver is a virtual reality (VR) application using a head-mounted display [13]. The surgeon can view the model and rotate and zoom as needed. Furthermore, transparency of different structures can be increased and decreased to allow for better visibility. All of the three aforementioned demonstration modes of a 3D reconstruction of the liver alleviate the surgeon of the task of reconstructing the CT scan mentally in ones’ head [14]. In addition all three modes facilitate the surgeon to share her or his thoughts with residents or students more easily enabling them to teach and supervise resection planning [15].

A previously conducted preclinical study compared the three modalities and found an improved visualization in 3D VR with head-mounted displays and 3D print [16]. In the current study, the clinical application of the described systems in a prospective randomized controlled trial is the focal point.

The interventions are compared to the current standard of viewing the CT scan on a regular computer screen. Therefore there is no placebo or sham group in action, which could potentially harm a patient. The regular CT scans are available to the surgical team at all times, during planning as well as during operation. The interventions act as supplement to the gold standard and have the potential to not only aid the surgeon but also the teaching of residents and students. The trial is expected to show, which demonstration modality of 3D models is helpful for liver resection planning and which demonstration mode facilitates the highest usability and best outcome. Generating a 3D model of the liver is a time-consuming task [7] and costs of 3D prints and VR head-mounted displays and personal computers capable of displaying VR content make economical deliberations necessary and obvious. The results of this study may help in the decision process, whether these techniques should and can be applied on a regular basis or only for well-chosen cases.

### Objectives {7}

The objective of the trial is to compare the three demonstration modes of a 3D reconstruction of the liver, being 3D PDF, 3D print and 3D VR using a head-mounted display, to the current gold standard of a 2D CT scan displayed by a regular computer screen. The main end-point is the comparison of planned resection volume (RLV1) and actual resection volume (RLV2) measured by water displacement volumetry ((RLV2-RLV1)/RLV1). Furthermore, patients’ characteristics as well as intraoperative outcome and postoperative morbidity and mortality will be assessed to ensure comparability of study groups. The surgical team will be presented a questionnaire to usability and handling of the demonstration modes.

### Trial design {8}

The study is designed as an exploratory prospective randomized trial with even allocation into all four study groups.

## Methods: participants, interventions, and outcomes

### Study setting {9}

The trial is planned as a monocentric study in the University hospital Mainz, Germany in the Department for General, Visceral and Transplant surgery. The department is among others specialized in hepatobiliary surgery with a case load of more than 300 liver resections each year.

### Eligibility criteria {10}

Inclusion criteria

- Elective primary conventional or laparoscopic liver resection
- Anatomical or atypical liver resection
- Major liver resection (>3 segments)
- Age >18 years
- Availability of a three-phase contrast-enhanced CT scan
- Written consent

Exclusion criteria

- Minor liver resection
- Recurrent liver resection
- Multi-visceral resection planned (i.e. simultaneous colon resection)
- Cirrhosis of the liver Child B or Child C

### Who will take informed consent? {26a}

The patients will be screened for eligibility in the outpatient clinic, where all patients of the department are presented for evaluation of resectability and preparation for operation. The researchers of the study team are trained physicians with experience in hepatobiliary surgery and will conduct the screening as well as the informed consent. Eligible patients will be offered the opportunity to participate and included or excluded based on their decision.

### Additional consent provisions for collection and use of participant data and biological specimens {26b}

The data collected include patient specifics as well as intraoperative and postoperative outcome data, patients are asked for written consent to recording and publishing these data anonymously. On the written consent form participants are informed, that they can withdraw from consent at any time. In case of withdrawal the data will not be used. There is no further data collection or obtainment of biological specimens in this study, hence this is not applicable.

## Interventions

### Explanation for the choice of comparators {6b}

Resection volume in liver surgery is a critical parameter correlating both to liver failure as well as postoperative morbidity. The presented trial aims to compare planned resection volume and actual resection volume throughout the aforementioned demonstrations modes to see which mode enables the most accurate prediction of resection volume. Furthermore, usability scores and handling of the different demonstration modes will be compared to determine, if one mode is preferable over the others. Patients’ characteristics as well as intraoperative outcome and postoperative morbidity and mortality are recorded for descriptive statistics and to unveil any imbalances of the study group. To compare patients’ general condition, the Charleson Comorbidity Index [17] is used; to assess postoperative complications, Dindo-Clavien Score and the comprehensive compication index are used.  (DOI: 10.1097/SLA.0000000000002902 [18]).

### Intervention description {11a}

After written consent, the patients are randomized into one of four groups using an envelope system. Randomization is not conducted by the researchers but by an impartial study nurse. Reconstruction of the preoperative CT scan into a 3D data set is performed by the researchers of the study group in the program Synapse 3D (Fujifilm Europe GmbH, Düsseldorf). In case of randomization into the 3D print group, the data set is anonymized and STL files are sent to a cooperating company (Cella Medical Solutions, Murcia, Spain) for printing. According to randomization, the 3D data set is then displayed to the surgeon assigned to the case not later than the day before surgery for resection planning. The surgeon is assisted by a member of the study group in terms of technical support and calculation of the planned resection volume (RLV1). At the end of surgery, the actual resection volume (RLV2) is determined by water displacement volumetry. A questionnaire about usability and subjective task load using the System Usability Scale (SUS) [19] and NASA Task Load Index (TLX) [20] is presented to the surgeons after operation. The data will be collected in a local database, to which only researchers of the study group have access. Data will be stored for 10 years according to the requirements of the local ethics committee.

### Criteria for discontinuing or modifying allocated interventions {11b}

All surgeons conducting liver resections in this study are senior surgeons with years of experience in hepatobiliary surgery. If a case is particularly demanding and the surgeon asks for another demonstration mode, this wish will be granted. During operation, the conventional 2D CT scans will be available to the surgeon, as well as the 3D print and the 3D PDF for the respecting groups. Due to technical restrictions, 3D VR using a head-mounted display will not be used in the operating room.

### Strategies to improve adherence to interventions {11c}

Adherence to intervention will be boosted by the technical support by the research group. Especially for surgeons, who have not used a VR head-mounted display before, help and guidance are necessary in the beginning. It is to be expected that the support needed will decrease during the course of the study.

### Relevant concomitant care permitted or prohibited during the trial {11d}

Not applicable to this trial. There are no restrictions regarding concomitant care during the trial. Using 3D PDF, 3D print or 3D VR in addition to regular cross-section imaging will not require alteration to usual care pathways for liver resection (including medication, drainages, sutures or imaging after surgery).

### Provisions for post-trial care {30}

There is no specific post-trial care. The patients are treated in the hospital for the time needed for them to recover. Most patients visit the out-patient clinic about two weeks after discharge for a regular check-up and about three months after surgery for regular tumour aftercare. The patients included in the study do not differ in this regard from other patients, so no special care is needed. There is no compensation for patients of any kind and there is no harm or higher risk of complication to be expected, since the method of operation is not altered.

### Outcomes {12}

Primary outcome is the quotient of planned resection volume (RLV1) and actual resection volume (RLV2) calculated as (RLV2-RLV1)/RLV1. The resection volume will be measured in millilitres; planned resection volume will be measured using one of the four demonstration modes and volumetry no late than the day before surgery. Actual resection volume will be measured the day of surgery. This parameter is critical to determine how accurate the predicted resection volume is between the different groups.

Secondary outcome consists of usability and subjective task load using SUS and the NASA TLX. A system is less likely to be used, if it is complicated or the controls are hard to remember or challenging to conduct. Thus these scores will show if implementation into daily clinical use is feasible for the different demonstration modes. The described systems are being tested in order to simplify liver resection planning and teaching, thus these measures are critical to determine usability and likelihood of daily use.

Furthermore, patients’ characteristics as well as intraoperative outcome (i.e. resection margin, blood loss, duration of surgery), pathological findings and postoperative morbidity and mortality are assessed in order to describe the study collective properly. Since liver resections can vary greatly in extent even in the chosen group of major resections, it is important to put patients’ characteristics and intraoperative and postoperative measures into perspective with the planned and actual surgical procedure.

### Participant timeline {13}

The participant timeline is shown in Table 1.

**Table 1:** Participant timeline

	Study period					
	Enrolment	Allocation	Post-allocation			
Timepoint	***− 1 day−0***	0	***+2 week***	***Day of surgery***	***POD 30***	***POD 90***
**Enrolment:**						
**Eligibility screen**	X					
**Informed consent**	X					
**Allocation**		X				
**Interventions:**						
***3D PDF***			X			
***3D print***			X			
***3D VR***			X			
***Control***						
**Assessments:**						
***Patients’ characterisctis***	X					
***Planned resection volume***			X			
***Preoperative questionnaire (planned resection)***			X			
***Postoperative questionnaire (SUS, NASA TLX, teaching experience)***				X		
***Actual resection volume***				X		
***Intraoperative specifics***				X		
***Pathological findings***					X	
***30-day morbidity (Dindo-Clavien)***					X	
***30-day mortality***					X	
***90-day mortality***						X

### Sample size {14}

Sample size was calculated in collaboration with the Institute of Medical Biostatistics, Epidemiology and Informatics (IMBEI) and the Interdisciplinary Center for Clinical Trials (IZKS), University Medical Center of the Johannes Gutenberg-University Mainz using G*Power (Düsseldorf, Germany) and a two-sided *t* test [21]. A power of 80% with a global significance level of 5% and a local significance level of 0.017 was chosen. Assuming a standard deviation of the mean of 20% in the control group and 10% in each 3D group, a number of 23 patients per group was calculated. Because of possible dropouts, we decided to increase the case number to 25 per group, deeming the total trial population *n* = 100.

### Recruitment {15}

Since the Department of General, Visceral and Transplant Surgery, University Medical Center of the Johannes Gutenberg-University is a certified nationwide centre of excellence in hepatobiliary surgery, patient clientele include all types of benign and malign liver resections. With a case load of about 300 liver resections per year it is deemed realistic to recruit participants within 2 years. Patients will be screened for eligibility in the outpatient clinic and if eligible offered participation on a voluntary basis.

## Assignment of interventions: allocation

### Sequence generation {16a}

After written consent and baseline assessment, patients will be randomly assigned to one of four groups at a ratio of 1:1. An envelope system utilizing opaque unmarked envelopes will be used for randomization. Randomization will be performed by an independent study nurse.

### Concealment mechanism {16b}

Randomization will be performed by an independent study nurse, who is blinded about the trial, using opaque, sealed and unmarked envelopes being drawn from a box without putting them back. Each participant will be represented in the database by a serial number.

### Implementation {16c}

The researchers of the study group will recruit participants and obtain written informed consent before inclusion into the trial. The participant is then reported to the study nurse using his or her unique serial number and the randomization is then performed by the study nurse.

## Assignment of interventions: blinding

### Who will be blinded {17a}

There is no blinding planned for this specific trial.

### Procedure for unblinding of needed {17b}

Not applicable since there is no blinding planned.

## Data collection and management

### Plans for assessment and collection of outcomes {18a}

The researchers of the study group will conduct the questionnaires and be responsible for data collection. The questionnaires, scores and indices (SUS, NASA TLX, Charlson Comorbidity Index and Dindo-Clavien Scores) used in this trial have been used and validated in prior studies [17–20]. Furthermore, senior surgeons and residents will be interviewed regarding their teaching respectively learning experience. Data will be collected in a local database using SPSS Statistics Version 27 (IBM, NY, USA).

### Plans to promote participant retention and complete follow-up {18b}

Since there is only a short follow-up period and no special action by the patients needed, there is no specific plan to facilitate the completion of follow-up. Data of patients who withdraw consent for the study, will not be used in analyses. If the surgeon in charge decides to take the patient out of their randomized group (i.e. in especially demanding cases) data will still be collected for an “as intended” analysis and in order to analyse all patients, who have been taken out of their randomized group.

### Data management {19}

The case report forms (CRF) with all patients’ characteristics, intraoperative and postoperative data and questionnaires are logged in paper format and stored in a locked closet in a locked room only accessibly for members of the research team. The CRF will be transferred to a local database for data analyzation using SPSS Statistics Version 27 (IBM, NY, USA). To promote data quality entries will be double-checked by a second researcher.

### Confidentiality {27}

The database, to which all of the CRF is transferred to, will only contain a unique number for each patient and no personal data. The unique number is logged in a separate database with personal data of each patient to facilitate follow-up, this database is only available to selected members of the study group. The paper forms of the CRF will be stored in a locked closet in a locked room, to which only the study group has access. The database containing the CRF will be stored locally and only be accessible to members of the research team as well.

### Plans for collection, laboratory evaluation and storage of biological specimens for genetic or molecular analysis in this trial/future sue {33}

Not applicable since there are no biological specimens collected.

## Statistical methods

### Statistical methods for primary and secondary outcomes {20a}

The primary analyses of the trial will adopt the per-protocol set (PPS), since there are only few dropouts to be expected. Patients withdrawing consent will not be included in analyses. Patients dropping out, due to a senior surgeons’ decision, will be analysed following the intention-to-treat (ITT) principle. The primary outcome, which is the quotient (RLV2-RLV1)/RLV1, will be analysed using the Wilcoxon Test. Continuous variables, as in the patients’ characteristics and perioperative and postoperative outcome, will be presented as mean ± standard deviation or median including interquartile range, depending on distribution. In order to compare the different study groups, an independent t-test will be applied. Variables with dichotomous values will be analysed using chi-square test.

### Interim analyses {21b}

There are no interim analyses at a fixed point in the study protocol planned. Since the intervention is not suspected to have an impact on patient safety, termination of the trial is not planned.

### Methods for additional analyses (e.g. subgroup analyses) {20b}

There are no additional analyses planned.

### Methods in analysis to handle protocol non-adherence and any statistical methods to handle missing data {20c}

Due to the short study period and the randomization not being affected by patients’ compliance or behaviour, missing data caused by non-adherence are not to be expected.

### Plans to give access to the full protocol, participant-level data and statistical code {31c}

Trial data will be available in the final publication. The full study protocol is accessible in the submitted manuscript.

## Oversight and monitoring

### Composition of the coordinating centre and trial steering committee {5d}

The trial steering committee will be composed of one principal investigator and two main investigators, in charge of administration and supervision of the study. Three physicians, who are part of the research group, will be responsible for conducting the trial in the coordinating centre, as well as data management. One study nurse will assist in the trial.

### Composition of the data monitoring committee, its role and reporting structure {21a}

There is no data monitoring committee planned to be in charge of the trial, since it is a monocentric study with low risk for patient safety.

### Adverse event reporting and harms {22}

Any adverse events (AE) or serious adverse events (SAE) will be monitored, recorded and in case of an SAE reported to the local ethical committee. In case of a suspected unexpected serious adverse reaction (SUSAR) report to the ethical committee will be made for reconsideration. Since the trial does not influence the method of operation and does not administer any study drugs, the occurrence of a SUSAR is deemed to be highly unlikely.

### Frequency and plans for auditing trial conduct {23}

A monthly supervision and discussion of study progress with the research group and at least one member of the steering committee is planned. Regular audits of the department include clinical trials.

### Plans for communicating important protocol amendments to relevant parties (e.g. trial participants, ethical committees9 {25}

All amendments and changes to the study protocol as well as changes in investigators will be reported to the local ethical committee right away. For trial specific communication with the local ethical committee, there is a two-step verification guarded online application available. Participants will be informed of changes by phone. Changes in time schedule that change the course of funding will be reported to the German Research Foundation.

### Dissemination plans {31a}

The results of this trial will be presented at relevant national and international conferences and the findings will be submitted for publication in peer-reviewed journals.

## Discussion

This study aims to explore the effect of different demonstration modes in planning of liver resections in order to provide surgeons with a tool to facilitate adequate planning in complex situations as well as to teach this important planning step to younger surgeons. The sample size for the study of 100 participants is expected to be attainable within two years in this high volume centre. However delayed inclusion can be seen in many clinical trials since the start of the COVID-19 pandemic, thus sufficient time to prolong recruiting, if necessary, is accounted for.

Group size of 25 patients in each group is sufficient for statistical analyses; however, it is possible that a skew between groups occurs due to very large tumours in need of extensive resection associated with higher perioperative risks. If applicable, a subgroup analysis with and without said outliers will be considered.

The trial is a first clinical test of resection planning in liver surgery in VR using a head-mounted display. Novel technologies usually raise interest at first but implementation in daily clinical use is a lengthy process, which is hardly predictable. Thus a lasting employment of VR using a head-mounted display for liver resection planning cannot be assumed. If implementation of this technology into daily practice is sustainably achieved and well accepted by users, expansion into different fields of operation planning is possible and desirable.

## Trial status

The presented manuscript contains version 4.0, dated to 15th of November 2021 of the study protocol. Recruiting has not yet started and is expected to start in April 2022. It is expected to finish after 24 months in April 2024.

## Supplementary Information

**Additional file 1.**

## Data Availability

The data acquired during the trial will be kept strictly confidential and only be accessible by members of the research group. Personal information of patients will not be released and their personal privacy will be protected.

## Abbreviations

AE: Sdverse event
CRF: Case report form
CT scan: Computer assisted tomography
ITT: Intention-to-treat
MRI: Magnetic resonance imaging
NASA TLX: National Aeronautics and Space Administration Task Load Index
PPS: Per-protocol set
RLV1: Planned resection volume
RLV2: Actual resection volume
SAE: Serious adverse event
SUSAR: Suspected unexpected serious adverse reaction
SUS: System Usability Scale
VR: Virtual reality
