# Supplementary figures and images for: Patient-individualized resection planning in liver surgery using 3D print and virtual reality (i-LiVR)—a study protocol for a prospective randomized controlled trial
Source: Trials. 2022 May 13;23:403. doi: 10.1186/s13063-022-06347-0 (PMC9100295; doi:10.1186/s13063-022-06347-0)

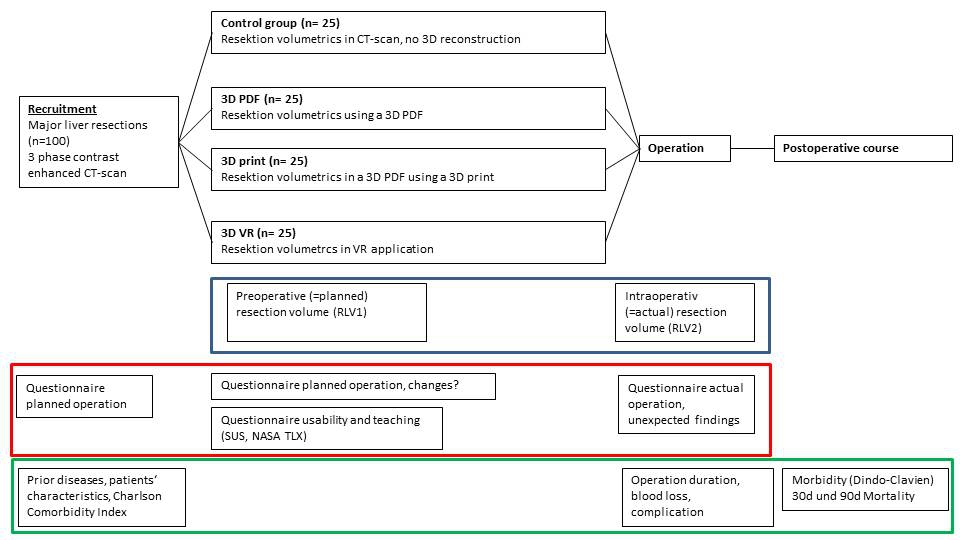

Supplement: Supplementary file 1 — Additional file 1. [file 13063_2022_6347_MOESM1_ESM.jpg]
